# Bariatric Surgery Induces a Differential Effect on Plasma Aldosterone in Comparison to Dietary Advice Alone

**DOI:** 10.3389/fendo.2021.745045

**Published:** 2021-10-05

**Authors:** Maxime Berney, Nima Vakilzadeh, Marc Maillard, Mohamed Faouzi, Eric Grouzmann, Olivier Bonny, Lucie Favre, Grégoire Wuerzner

**Affiliations:** ^1^ Service of Nephrology and Hypertension, Department of Medicine, Lausanne University Hospital and University of Lausanne, Lausanne, Switzerland; ^2^ Département de Formation, Recherche et Innovation, Unisanté, University of Lausanne, Lausanne, Switzerland; ^3^ Laboratoire des Catécholamines et Peptides, Service de Biomédecine, Lausanne University Hospital and University of Lausanne, Lausanne, Switzerland; ^4^ Department of Biomedical Sciences, University of Lausanne, Lausanne, Switzerland; ^5^ Service of Endocrinology, Diabetes and Metabolism, Lausanne University Hospital, Lausanne, Switzerland

**Keywords:** bariatric-surgery, obesity, hypertension, renin-angiotensin-aldosterone system, sodium excretion

## Abstract

**Background and Objectives:**

The pathophysiological mechanisms linking weight loss to blood pressure (BP) reduction are not completely understood. The objective of this study was to compare the effect of weight loss after Roux-en-Y gastric bypass (RYGB) on BP, renin-angiotensin-aldosterone system (RAAS), and urinary electrolytes excretion to those of dietary advice.

**Methods:**

This was a case-control prospective study including obese patients referred for RYGB (cases) and obese receiving diet advice only (controls). Ambulatory BP, plasma renin activity (PRA), plasma aldosterone concentration (PAC), and urinary electrolytes were measured before (M0) and after intervention (M3: 3 months and M12: 12 months).

**Results:**

Twenty-five patients were included in the RYGB group and twelve patients in the control group. After 12 months, weight loss (-42 ± 11.5 *vs* -12.3 ± 6.3 kg in the control group, p=0.001) and decrease in PAC were more pronounced in the RYGB group (-34 ± 76 *vs* +14 ± 45 pg/ml in the control group, p=0.002). There was no difference in PRA between both groups (-0.08 ± 1.68 *vs* 0.01 ± 0.37 ng/ml/h, p=0.31). Sodium excretion was more marked in the RYGB group after 3 months only (-89 ± 14.9 *vs* -9.9 ± 27.9 mmol/day, p=0.009). The decrease in SBP was similar between both groups (-6.9 ± 9.9 *vs* -7.1 ± 11.9 mmHg in the control group, p=0.96).

**Conclusions:**

Bariatric-induced weight loss induces a progressive decrease in PAC independently of PRA and sodium excretion. Whether this decrease in PAC affects target organ damage in the long term remains to be determined.

**Clinical Trial Registration:**

ClinicalTrials.gov, identifier NCT02218112.

## Introduction

Hypertension and obesity are two highly prevalent diseases with a major cardiovascular burden (1, 2). According to data from the Framingham Heart Study, the risk for hypertension directly related to weight excess is about 80% in men and 65% in women (3). Many studies have demonstrated that obesity is an independent risk factor for hypertension (4).

Potential mechanisms linking obesity to hypertension are many and growing evidence suggests the implication of the renin-angiotensin-aldosterone system (RAAS) in the pathogenesis (5). Excess visceral adipose tissue is closely related to the development of hypertension (6, 7). In addition, plasma aldosterone concentration (PAC) is associated with insulin resistance and visceral adiposity (8). White adipocytes also secrete adipokines, sometimes called aldosterone-releasing factors that can stimulate aldosterone secretion from adrenal cells (9, 10). According to some epidemiological studies, obese patients often have a mild to moderate increase of the components of the RAAS (5, 11–14). Other reports showed that only PAC and not plasma renin activity (PRA) was elevated and associated with obesity and hypertension (15, 16).

Among weight loss-inducing intervention, bariatric surgery remains the most successful approach for sustained weight loss. Most studies have shown a significant improvement in blood pressure control after bariatric surgery (17–23). However, the effects of bariatric surgery-induced weight loss on the different components of the RAAS have been conflicting (12, 22–30). In addition, the effects of Roux-en-Y gastric bypass (RYGB) on blood pressure compared to diet alone have not been investigated simultaneously.

Our primary objective was to evaluate the effect of weight loss on PAC and PRA after RYGB and to compare it to weight loss induced by diet alone. In addition, we examined the effect of RYGB-related weight loss on blood pressure and possible confounding factors such as adipokines, sodium excretion and measured glomerular filtration rate (mGFR).

## Methods

### Population and Study Design

This was a prospective observational study comparing the effect of RYGB to diet alone on BP, PRA, PAC, adipokines and urinary electrolytes. Obese patients with body mass index >35 kg/m^2^ referred to the obesity outpatient clinic of the Lausanne University Hospital for bariatric surgery or for nutritional advice alone could be included in the study. Patients who fulfilled criteria for RYGB but refused the surgical intervention were included in the control group. The surgical technique was standardized, and all patients had RYGB with a small gastric pouch (< 25 ml), an alimentary limb of 100 cm and a biliopancreatic limb of 50 cm. Patients referred for bariatric surgery were assessed and prepared by a multidisciplinary team and fulfilled the eligibility criteria for undergoing this intervention. Control patients benefited from conservative treatment with multicomponent approach comprising dietary change, behavioral therapy and motivational intervention to increase physical activity over the one-year study period. They did not receive pharmacotherapies for weight loss. Exclusion criteria included any acute illness, asthma, orthostatic hypotension, chronic treatment with drugs affecting kidney function such as non-steroidal anti-inflammatory drugs, renal artery stenosis, renal transplantation, polycystic kidney disease, primary and secondary aldosteronism, and chronic liver disease.

Patients were investigated on three separate occasions: one baseline visit (M0) and two follow-up visits, 3 (M3) and 12 (M12) months after the interventions (RYGB or dietary advice alone).

### Data Collection

Each visit included a 24h urine collection (for measurement of electrolytes excretion and albuminuria) and a 24h ambulatory blood pressure measurement (ABPM) with an adequately sized cuff (WatchBP03, Microlife AG, 9443 Widnau, Switzerland), which were started the day before the study day. BP measurements during ABPM were taken every 20 min in the daytime and every 30 min at night. Daytime and nighttime periods were defined according to patients’ diary (bedtime and wakeup time). Daytime ABPM and nighttime ABPM were discarded if the number of readings were respectively less than 20 and 7 measurements.

On the study day, participants arrived at Clinic at 8.00 am. Weight, office blood pressure and heart rate were measured. A venous catheter was inserted into one of the antecubital veins and a bolus of inulin followed by a constant infusion was given to every participant to measure glomerular filtration rate (mGFR). An initial oral water load (400 ml) was given, followed by 150 ml of water hourly to insure constant urine output. Two one-hour urine collections were performed from 10 am to 12 pm to allow the calculation of inulin clearance according to the single compartment model. PAC, PRA, leptin and adiponectin concentrations were measured in the recumbent position at 11 am.

24-hour urinary electrolyte excretion rate was calculated as Ux ·V (µmol/min) and clearances (ml/min) were calculated using the standard formula Cx = Ux ·V/Px where Ux and Px are the urine and plasma concentrations of x and V is the urine flow rate in ml/min.

PRA, PAC, plasma leptin and adiponectin were measured with commercial radioimmunoassay or sandwich ELISA (31). Plasma catecholamine (norepinephrine and epinephrine) were measured using ultra high-performance liquid chromatography-tandem mass spectrometry (32).

In patients with hypertension, calcium channel blockers were the only antihypertensive drugs allowed during the study day. Other antihypertensive drugs were stopped 48 hours before ABPM was started (washout period). Spironolactone was stopped 6 weeks before the study days. Investigations were interrupted for safety reason if BP was >180/110 mmHg during the 48 hours before the study days, when antihypertensive drugs other than calcium channel blockers were withdrawn.

Central obesity was defined as a waist-to-hip ratio of 0.90 or more in men and of 0.85 or more in women and peripheral obesity as a waist-to-hip ratio of less than 0.90 in men and of less than 0.85 in women. Glomerular hyperfiltration was defined as mGFR>140 ml/min (33, 34).

### Statistical Analysis

Statistical analyses were performed using Stata software (*StataCorp. 2019. Stata Statistical Software: Release 16. College Station, TX: StataCorp LLC*). Data were summarized by group (Control *vs* RYGB) and by phase (M0, M3, M12) and expressed as mean ± standard deviation (SD) for normally distributed data and as median and interquartile range (IQR) for non- normally distributed data. To evaluate the effect of RYGB compared to diet alone on different outcomes (daytime systolic and diastolic blood pressure, weight, BMI, PAC, plasma renin activity, epinephrine, norepinephrine, adiponectin, leptin, albuminuria (24-hour urinary collection) and urinary electrolytes excretion), a linear mixed-effects model was used. Non-normally distributed outcomes were first log-transformed and analyzed on the logarithmic scale. Tested covariates include group (RYGB *vs* Control), phase (M0, M3, M12), their interaction group * phase, age, sex and height. Results were presented for univariate analysis and adjusted analysis when the group effect changed by adjusting for other covariates.

## Results

Thirty-seven obese patients (25 RYGB and 12 controls) were included in the study. All patients in the RYGB group attended the three visits (baseline, 3 months and 12 months). Two patients in the control group did not attend the third visit because they were referred for RYGB surgery before the last scheduled visit.

The baseline characteristics of the participants are shown in **Table 1**. No significant differences in age, weight, BMI, office BP and between obesity types were observed between both groups. Note that only daytime ABPM is shown because the quality of nighttime ABPM did not reach the requirements. Based on baseline daytime ABPM, 20% had hypertension (daytime BP ≥ 135/85 mmHg) in the RYGB group and 33% in the control group. Antihypertensive medications were used by 9 patients (24%), including 6 patients in the RYGB group and 3 patients in the control group.

**Table 1:** Baseline characteristics of the study participants.

Characteristics	RYGB (n=25)	Controls (n=12)	P-value
**Age (years)**	42 ± 10	48 ± 14	0.51
**Female sex (%)**	72	58	0.42
**Weight (kg)**	121 ± 19	126 ± 23	0.46
**BMI (kg/m^2^)**	43 ± 5.3	43 ± 5.1	0.87
**Office SBP (mmHg)**	120 ± 3	123 ± 3	0.42
**Office DBP (mmHg)**	79 ± 2	83 ± 3	0.27
**Central obesity**^**a**^ **(%)**	52	50	0.43

Values are presented as mean (SD) or percentages. RYGB, Roux-en-Y gastric bypass; BMI, body mass index; SBP, systolic blood pressure; DBP, diastolic blood pressure.

a Defined by a waist-hip ratio >0.9 in men or >0.85 in women.

### Changes in Weight and BMI

**Figure 1:**
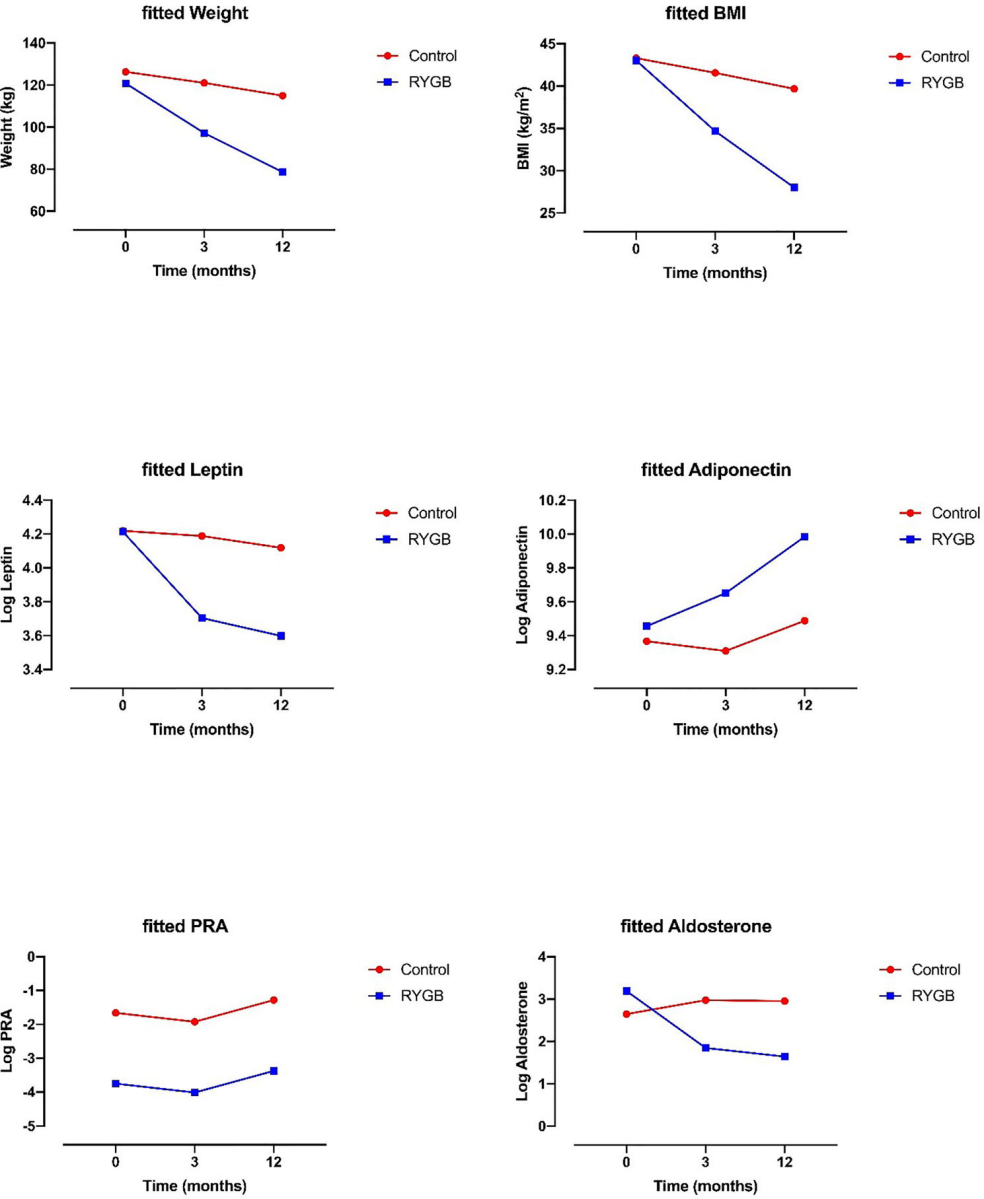
Effect of RYGB on weight, BMI, leptin, adiponectin, plasma renin activity, and aldosterone. Data on graphs are fitted from linear mixed model analysis.

Weight loss was -23.6 ± 8.5 kg (-19.4 ± 5.3 percent of total body weight loss, %TWL) at 3 months and -42.2 ± 11.5 kg (-34.8 ± 6.9%TWL) at 12 months in the RYGB group and -5.3 ± 8.2 kg (-3.8 ± 5.9%TWL) after 3 months and -12 ± 6.3 kg (-8.3 ± 13.6%TWL) after 12 months in the control group. There was a significant interaction between time and groups, with the RYGB group losing more weight than the control group for one year (**Table 2** and **Figure 1**).

**Table 2:** Changes in blood pressures, heart rate, weight, BMI, PRA, PAC, epinephrine, norepinephrine, and adipokines over time.

	Groups	M0 (baseline) mean ± sd	M3 mean ± sd	M12 mean ± sd	Univariate analysis Group β (p-value)	Univariate analysis Phase β (p-value)	Adjusted analysis β (p-value)	Adjusted analysis Phase X Group β (p-value)
**Daytime systolic blood pressure (mmHg)**	Control	126 ± 9	122 ± 9	120 ± 6	-5.32 (p=0.014)	**M3 *vs* M0** -1.79 (p=0.36) **M12 *vs* M0** -6.79 (p=0.001)	**M3 *vs* M0** -3.89 (p=0.26) **M12 *vs* M0** -5.82 (p=0.12) **Group** -5.74 (p=0.07)	**M3 *vs* M0** *No interaction* **M12 *vs* M0** *No interaction*
	RYGB	120 ± 8	120 ± 10	114 ± 8				
**Daytime diastolic blood pressure (mmHg)**	Control	82 ± 5	79 ± 7	76 ± 7	-4.36 (p=0.028)	**M3 *vs* M0** -0.67 (p=0.64) **M12 *vs* M0** -4.41 (p=0.002)	**M3 *vs* M0** -3.3 (p=0.19) **M12 *vs* M0** -6.4 (p=0.02) **Group** -6.25 (p=0.02)	**M3 *vs* M0** *No interaction* **M12 *vs* M0** *No interaction*
	RYGB	76 ± 6	76 ± 8	73 ± 8				
**Heart rate (bpm)**	Control	86 ± 8	85 ± 9	80 ± 11	-6.45 (p=0.016)	**M3 *vs* M0** -1.69 (p=0.32) **M12 *vs* M0** -5.76 (p=0.001)	**M3 *vs* M0** -0.41 (p=0.89) **M12 *vs* M0** -4.85 (p=0.13) **Group** -5.07 (p=0.13)	**M3 *vs* M0** *No interaction* **M12 *vs* M0** *No interaction*
	RYGB	81 ± 8	78 ± 9	75 ± 10				
**Weight (kg)**	Control	126 ± 23	121 ± 21	116 ± 21	-22.65 (p=0.001)	**M3 *vs* M0** -17.7 (p<0.001) **M12 *vs* M0** -33.61 (p<0.001)	**M3 *vs* M0** -5.25 (p=0.06) **M12 *vs* M0** -11.37 (p<0.001) **Group** -5.43 (p=0.38)	**M3 *vs* M0** -18.4 (p=0.0001) **M12 *vs* M0** -30.9 (p=0.0001)
	RYGB	121 ± 19	97 ± 15	78 ± 14				
**BMI (kg/m^2^)**	Control	43 ± 5	42 ± 5	39 ± 5	-6.36 (p<0.001)	**M3 *vs* M0** -6.2 (p<0.001) **M12 *vs* M0** -11.86 (p<0.001)	**M3 *vs* M0** -1.73 (p=0.054) **M12 *vs* M0** -3.63 (p<0.001) **Group** -0.27 (p=0.87)	**M3 *vs* M0** -6.62 (<0.001) **M12 *vs* M0** -11.4 (<0.001)
	RYGB	43 ± 5	35 ± 5	28 ± 4				
**PAC (pg/ml)**	Control	42 ± 23	52 ± 40	57 ± 40	-0.62 (p=0.12)	**M3 *vs* M0** -0.8 (p=0.004) **M12 *vs* M0** -1.02 (p<0.001)	**M3 *vs* M0** 0.33 (p=0.47) **M12 *vs* M0** 0.31 (p=0.54) **Group** 0.54 (p=0.28)	**M3 *vs* M0** -1.67 (p=0.002) **M12 *vs* M0** -1.85 (p=0.002)
	RYGB	71 ± 89	37 ± 43	36 ± 49				
**PRA (ng/ml/h)**	Control	0.3 ± 0.5	0.3 ± 0.8	0.5 ± 0.5	-2.05 (p=0.001)	**M3 *vs* M0** -0.26 (p=0.54) **M12 *vs* M0** 0.33 (p=0.45)	**M3 *vs* M0** 0.37 (p=0.74) **M12 *vs* M0** 1.06 (p=0.19) **Group** -1.48 (p=0.07)	**M3 *vs* M0** *No interaction* **M12 *vs* M0** *No interaction*
	RYGB	0.1 ± 0.2	0.1 ± 0.4	0.1 ± 0.2				
**Epinephrine (nmol/l)**	Control	0.07 ± 0.03	0.08 ± 0.09	0.11 ± 0.14	-0.12 (p=0.49)	**M3 *vs* M0** -0.08 (p= 0.59) **M12 *vs* M0** 0.24 (p=0.1)	**M3 *vs* M0** -1.98 (p=0.42) **M12 *vs* M0** 0.06 (p=0.81) **Group** -0.27 (p=0.25)	**M3 *vs* M0** *No interaction* **M12 *vs* M0** *No interaction*
	RYGB	0.06 ± 0.05	0.06 ± 0.04	0.09 ± 0.06				
**Norepinephrine (nmol/l)**	Control	1.6 ± 0.8	1.4 ± 0.6	1.15 ± 0.7	-0.14 (p=0.43)	**M3 *vs* M0** -0.18 (p=0.18) **M12 *vs* M0** -0.34 (p=0.009)	**M3 *vs* M0** -1.18 (p=0.41) **M12 *vs* M0** -0.49 (p=0.04) **Group** -0.18 (p=0.43)	**M3 *vs* M0** *No interaction* **M12 *vs* M0** *No interaction*
	RYGB	1.4 ± 0.9	1.2 ± 0.5	1.1 ± 0.6				
**Leptin (mcg/l)**	Control	51 ± 23	49 ± 22	41 ± 18	-0.34 (p=0.001)	**M3 *vs* M0** -0.35 (p<0.001) **M12 *vs* M0** -0.47 (p<0.001)	**M3 *vs* M0** -0.031 (p= 0.69) **M12 *vs* M0** -0.1 (p=0.24) **Group** -0.0056 (p=0.96)	**M3 *vs* M0** -0.48 (<0.001) **M12 *vs* M0** -0.51 (<0.001)
	RYGB	51 ± 25	22 ± 14	18 ± 16				
**Adiponectin (mcg/ml)**	Control	9.5 ± 3.3	8.9 ± 3.5	11.7 ± 4.9	0.32 (p=0.01)	**M3 *vs* M0** 0.11 (p=0.062) **M12 *vs* M0** 0.41 (p<0.001)	**M3 *vs* M0** -0.57 (p=0.56) **M12 *vs* M0** 0.12 (p=0.27) **Group** 0.09 (p=0.63)	**M3 *vs* M0** 0.25 (p=0.036) **M12 *vs* M0** 0.41 (p=0.002)
	RYGB	11.4 ± 5.6	14.7 ± 8.6	20.8 ± 9.1				

BMI, Body mass index; PAC, plasma aldosterone concentration; PRA, plasma renin activity; M0, baseline (Month 0); M3, third month; M12, 12th month.

BMI decreased by -8.3 ± 2.5 kg/m^2^ at 3 months and by -15 ± 3.6 kg/m^2^ after 12 months. There was a significant interaction between time and groups, with a decrease in BMI that was more pronounced in the RYGB group for one year (**Table 2** and **Figure 1**).

### Changes in Daytime Blood Pressure

Changes in daytime systolic blood pressure (SBP) were -0.9 ± 8.7 mmHg in the RYGB group and -3.6 ± 17.5 mmHg in the control group 3 months after intervention. Change in daytime diastolic blood pressure (DBP) was +0.4 mmHg ± 7.4 mmHg in the RYGB group and -3.2 ± 10.6 mmHg in the control group at 3 months. One year after the intervention, change in daytime SBP was -6.9 ± 9.9 mmHg in the RYGB group and -7.1 ± 11.9 mmHg in the control group. Change in daytime DBP one year after the intervention was -3.8 ± 6.6 mmHg in the RYGB group and -7.4 ± 8.8 mmHg in the control group. Univariate analysis showed that daytime SBP and DBP were lower in the RYGB group compared to the control group and decreased 12 months after the intervention compared to baseline measures. In multivariate analysis, there was no significant interaction observed between time and groups after 3 or 12 months (**Table 2**).

### Changes in Plasma Renin Activity and Plasma Aldosterone

Univariate and adjusted analyses showed that PRA was lower in the RYGB group compared to the control group. There was no effect of time on PRA, nor a significant effect between groups and time. 3 months after the intervention, PAC decreased by -33.7 ± 95 pg/ml in the RYGB group and increased by +10.4 ± 36 pg/ml in the controls. After one year, change in PAC was -34.3 ± 76 pg/ml in the RYGB group and +14.2 ± 45 pg/ml in the controls. There was no difference between groups in PAC, but there was a progressive decrease after intervention at 3 and 12 months in the RYGB. A significant interaction was also observed between time and groups after 3 and 12 months with a greater decrease in PAC in the RYGB group (**Table 2** and **Figure 1**).

### Changes in Epinephrine and Norepinephrine

Univariate analysis and adjusted analyses showed a significant time effect for norepinephrine after 12 months that was lower after 12 months in both groups (**Table 2**). There was no significant group effect in univariate or adjusted analysis for those two parameters. Finally, no significant interaction was observed between time and groups after 3 or 12 months for both epinephrine and norepinephrine.

### Changes in Leptin and Adiponectin

3 months after the intervention, leptin decreased by -29.1 ± 17.4 mcg/l in the RYGB group and -2.1 ± 22.1 mcg/l in the control group. After 12 months, leptin decreased by -32.8 ± 17.9 mcg/l in the RYGB group and -3.4 ± 24 mcg/l in the controls. After 3 months and 12 months, adiponectin increased respectively by + 3.27 ± 6.2 mcg/ml and + 9.3 ± 8.4 mcg/ml in the RYGB and decreased by -0.6 ± 1.8 mcg/ml at 3 months and then increased by 1.9 ± 3.5 mcg/ml at 12 months in the controls.

Univariate analysis and adjusted analysis showed that there was a significant group and time effect for both leptin and adiponectin. A significant interaction was observed between time and groups with a greater decrease in leptin and a greater increase in adiponectin in the RYGB after 3 and 12 months (**Table 2** and **Figure 1**).

### Changes in Creatinine Clearance, Inulin Clearance, Albuminuria, and Electrolytes Excretion

3 months after the intervention, univariate analysis showed that there was a significant group and time effect for sodium, potassium, urate, calcium, phosphate and urea excretion (**Table 3**). Adjusted analysis confirmed the effect of time and group for these parameters after 3 months. A significant interaction was also observed between time and groups with a greater decrease in sodium, potassium, urate, calcium, phosphate, and magnesium and urea daily excretion in the RYGB after 3 months but not at 12 months.

**Table 3:** Changes in urinary electrolytes excretion, albuminuria, and renal clearances over time.

	Groups	M0 (baseline) mean ± sd	M3 mean ± sd	M12 mean ± sd	Univariate analysis Group β (p-value)	Univariate analysis Phase β (p-value)	Adjusted analysis β (p-value)	Adjusted analysis Phase X Group β (p-value)
**Sodium (mmol/24h)**	Control	207 ± 116	193 ± 93	157 ± 75	-60.1 (p=0.006)	**M3 *vs* M0** -65.5 (p<0.001) **M12 *vs* M0** -45.1 (p=0.001)	**M3 *vs* M0** -13.9 (p=0.5) **M12 *vs* M0** -36.6 (p=0.11) **Group** -29.34 (p=0.26)	**M3 *vs* M0** -77.1 (p=0.002) **M12 *vs* M0** *No interaction*
	RYGB	178 ± 85	87 ± 49	129 ± 46				
**Potassium (mmol/24h)**	Control	62 ± 29	62 ± 33	53 ± 20	-13.9 (p=0.016)	**M3 *vs* M0** -20.1 (p<0.001) **M12 *vs* M0** -13.9 (p=0.003)	**M3 *vs* M0** -0.98 (p=0.89) **M12 *vs* M0** -11.3 (p=0.17) **Group** -2.51 (p=0.75)	**M3 *vs* M0** -28.6 (p=0.002) **M12 *vs* M0** *No interaction*
	RYGB	60 ± 23	31 ± 12	46 ± 18				
**Urate (mmol/24h)**	Control	5.24 ± 1.24	4.47 ± 1.7	3.9 ± 1.12	-0.49 (p<0.001)	**M3 *vs* M0** -0.5 ± 0.07 (p<0.001) **M12 *vs* M0** -0.48 ± 0.08 (p<0.001)	**M3 *vs* M0** -0.21 (p=0.09) **M12 *vs* M0** -0.32 (p=0.018) **Group** -0.27 (p=0.037)	**M3 *vs* M0** -0.04 (p=0.004) **M12 *vs* M0** *No interaction*
	RYGB	4.24 ± 1.6	2.23 ± 0.7	2.6 ± 1				
**Calcium (mmol/24h)**	Control	5.6 ± 3.7	4.9 ± 2.5	4.2 ± 1.5	-0.2 (p=0.096)	**M3 *vs* M0** -0.23 (p=0.003) **M12 *vs* M0** -0.19 (p=0.016)	**M3 *vs* M0** -0.014 (p=0.92) **M12 *vs* M0** -0.1 (p=0.48) **Group** -0.3 (p=0.15)	**M3 *vs* M0** -0.33 (p=0.043) **M12 *vs* M0** *No interaction*
	RYGB	4.9 ± 2.7	2.9 ± 1.5	3.5 ± 2				
**Phosphate (mmol/24h)**	Control	37 ± 19	30 ± 10	30 ± 13	-0.23 (p=0.001)	**M3 *vs* M0** -0.3 (p<0.001) **M12 *vs* M0** -0.19 (p<0.001)	**M3 *vs* M0** -0.88 (p=0.52) **M12 *vs* M0** -0.84 (p=0.32) Group -0.7 (p=0.42)	**M3 *vs* M0** -0.31 (p=0.001) **M12 *vs* M0** *No interaction*
	RYGB	31 ± 12	14 ± 7	20 ± 8				
**Magnesium (mmol/24h)**	Control	4.5 ± 2.29	4 ± 1.8	3.9 ± 1.9	-0.15 (p=0.049)	**M3 *vs* M0** -0.17 (p=0.001) **M12 *vs* M0** 0.03 (p=0.62)	**M3 *vs* M0** -0.06 (p=0.52) **M12 *vs* M0** -0.63 (p=0.52) **Group** -0.13 (p=0.18)	**M3 *vs* M0** *No interaction* **M12 *vs* M0** *No interaction*
	RYGB	3.6 ± 1.7	2.4 ± 1	3.9 ± 2				
**Urea (mmol/24h)**	Control	361 ± 157	310 ± 134	296 ± 95	-118.8 (p<0.001)	**M3 *vs* M0** -122.9 (p<0.001) **M12 *vs* M0** -84.1 (p<0.001)	**M3 *vs* M0** -52.5 (p=0.08) **M12 *vs* M0** -60.7 (p=0.06) **Group** -69.3 (p=0.04)	**M3 *vs* M0** -106.1 (p=0.004) **M12 *vs* M0** *No interaction*
	RYGB	292 ± 97	133 ± 60	201 ± 63				
**Oxalate (µmol/24h)**	Control	353 ± 90	352 ± 98	303 ± 90	-0.22 (p=0.07)	**M3 *vs* M0** -0.05 (p=0.67) **M12 *vs* M0** 0.14 (p=0.26)	**M3 *vs* M0** -0.3 (p=0.89) **M12 *vs* M0** -0.22 (p=0.32) **Group** -0.38 (p=0.04)	**M3 *vs* M0** *No interaction* **M12 *vs* M0** 0.52 (p=0.045)
	RYGB	287 ± 123	265 ± 8	391 ± 257				
**Citrate (mg/24h)**	Control	585 ± 251	596 ± 424	511 ± 199	0.11 (p=0.36)	**M3 *vs* M0** 0.06 (p=0.45) **M12 *vs* M0** -0.003 (p=0.98)	**M3 *vs* M0** -0.61 (p=0.65) **M12 *vs* M0** -0.14 (p=0.34) **Group** -0.2 **(p=0.89)**	**M3 *vs* M0** *No interaction* **M12 *vs* M0** *No interaction*
	RYGB	634 ± 339	683 ± 298	692 ± 530				
**Albuminuria (mg/24h)**	Control	24.7 ± 36.1	17.5 ± 22.4	7.2 ± 3.2	-8.44 (p=0.04)	**M3 *vs* M0** -3.31 (p=0.37) **M12 *vs* M0** -5.09 (p=0.18)	**M3 *vs* M0** -6.78 (p=0.29) **M12 *vs* M0** -15.5 (p=0.03) **Group** -14.31 (p=0.02)	**M3 *vs* M0** *No interaction* **M12 *vs* M0** *No interaction*
	RYGB	9.9 ± 6.9	7.9 ± 4.8	8.8 ± 17.2				
**Creatinine clearance** **(ml/min)**	Control	159 ± 36	158 ± 55	133 ± 34	-0.28 (p=0.01)	**M3 *vs* M0** -0.16 (p=0.005) **M12 *vs* M0** -0.24 (p<0.001)	**M3 *vs* M0** -0.6 (p=0.55) **M12 *vs* M0** -0.18 (p=0.1) **Group** -0.19 (p=0.14)	**M3 *vs* M0** *No interaction* **M12 *vs* M0** *No interaction*
	RYGB	137 ± 53	113 ± 28	112 ± 40				
**Inulin clearance** **(ml/min)**	Control	111 ± 32	90 ± 48	89 ± 21	-10.17 (p=0.3)	**M3 *vs* M0** -23.7 (p<0.001) **M12 *vs* M0** -36.8 (p<0.001)	**M3 *vs* M0** -22.4 (p=0.018) **M12 *vs* M0** -30.8 (p=0.002) **Group** -3.98 (p=0.74)	**M3 *vs* M0** *No interaction* **M12 *vs* M0** *No interaction*
	RYGB	106 ± 42	83 ± 36	68 ± 20				

BMI, Body mass index; PAC, plasma aldosterone concentration; PRA, plasma renin activity; M0, baseline (Month 0); M3, third month; M12, 12th month.

In univariate analysis, no difference in oxalate excretion was detected between phases or groups. There was however a significant interaction between time and groups, with a greater excretion of oxalate after 12 months in the RYGB (**Table 3**).

After 3 months, creatinine clearance decreased by -24 ± 35 ml/min in the RYGB group and -1 ± 54 ml/min in the control group. After one year, creatinine clearance decreased by -25 ± 42 ml/min in the RYGB group and -26 ± 27 ml/min in the control group. In univariate and adjusted analysis there was a significant effect of group and time with lower creatinine clearance in the RYGB group and lower creatinine clearance after 3 and 12 months. There was however no significant interaction between groups and time (**Table 3**). Inulin clearance decreased by -23 ± 36 ml/min in the RYGB and by -21 ± 33 ml/min in the controls after 3 months. After 12 months, inulin clearance decreased by -38 ± 34 ml/min and by -22 ± 26 ml/min compared to baseline in the RYGB and controls, respectively. Univariate and adjusted analyses showed that there was a significant group and time effect but no interaction between group and time (**Table 3**). Twenty-four-hour albuminuria decreased in both groups. Univariate analysis showed no group of time effect. Adjusted analyses showed that there was a significant group and time effect after 12 months but no significant interaction between group and time (**Table 3**).

## Discussion

Our results show that compared to diet, weight loss is more pronounced after 12 months in the RYGB group, which is associated with a progressive decrease in PAC independently of sodium excretion and PRA. The time-dependent changes in leptin and adiponectin also showed difference between groups with a rapid decrease in leptin and a progressive increase in adiponectin in the RYGB compared to controls. In addition, sodium, potassium, calcium, phosphate, urate, urea and magnesium excretion decreased more during the first 3 months in the RYGB group as opposed to oxalate excretion, which increased in the RYGB at 12 months.

### Effects on the Renin-Angiotensin-Aldosterone and the Sympathetic Nervous System

Several epidemiological studies have shown that PAC is increased in obese patients supposedly following an activation of the RAAS and the sympathetic nervous system (3, 9, 12, 16). Some studies have demonstrated that both PAC and PRA decreased after weight loss suggesting that obesity may be associated with a systemic stimulation of the renin-angiotensin-aldosterone system (12, 22, 23, 25). However, other studies have shown that PAC decreased after weight loss while PRA remained stable (24, 26, 28, 30). The reasons behind those different observations remain unclear but one could be the existence of a local RAAS in adipose tissue, by opposition to the systemic RAAS (5). Indeed, it has been shown that adipocytes can produce aldosterone in an extra-adrenal and renin-independent pathway (9, 10). This adipocyte-derived aldosterone production does not appear to be influenced by usual hemodynamic RAAS feedback or salt load and might be the major explanation of the excess of PAC observed in the obese population. Studies have demonstrated that bariatric surgery-induced weight loss is associated with a massive reduction in visceral fat mass that is greater than reduction observed after diet-induced weight loss (35, 36).

We found that PRA was lower in the RYGB group throughout the study and remained stable in both groups during follow-up while PAC progressively decreased in the RYGB group. The lower values of PRA in the RYGB group could have been explained by an increase in sodium intake but this was not observed at baseline, nor was it at 3 months, when sodium excretion was distinctly decreased. Moreover, at 12 months, sodium excretion tended to return to baseline levels in the RYGB group, but PRA did not change, and PAC remained lower suggesting that PAC production was independent of salt intake and systemic PRA. In addition, protocol specific features such as water load before hormone measurement could explain low renin levels despite reduction in sodium intake. Serum potassium, another determinant of aldosterone secretion, did not change significantly during the study. Therefore, the decrease in PAC was also independent of serum potassium. Finally, the reduction of the initial obesity-associated hyperfiltration state to a normal or decreased GFR may also have played a role by decreasing sodium excretion and thus inhibiting renin secretion. Overall, these findings suggest a renin-independent effect of RYGB-associated weight loss on PAC secretion mediated by drastic reduction of the adipocytes mass after the surgery.

### Effects on Adipokines

In the present study, our findings are directly in line with previous findings, as we observed that bariatric-induced weight loss is associated with progressive rise in serum adiponectin level and a rapid fall of serum leptin. Indeed, it has been described that circulating leptin decreases by 50% during the first week after RYGB surgery, before significant weight loss, and tends to decrease further throughout the weight loss process (37). The marked fall of serum leptin concentration at very short term following RYGB is likely due to the postoperative temporary break of food intake (38, 39).

Previous studies have shown that leptin decreases after diet-induced weight loss and remains low during weight maintenance (40). In the control group, we observed a smaller but persistent leptin decrease as described in previous studies. Leptin seems to play a role in obesity-associated cardiovascular risk through the activation of the sympathetic nervous system (41, 42). We found that despite a major decrease in plasma leptin levels after RYGB, epinephrine and norepinephrine remained stable suggesting a preponderant role of other mechanisms in maintaining sympathetic system activation in the obese population. Moreover, our results show a parallel decrease in leptin and PAC. These findings are in line with the hypothesis of a bi-directional cross-talk between adipose tissue and adrenal glands (“fat-adrenal endocrine axis”) described by Infante & colleagues (43, 44). Indeed, white adipocytes secrete leptin, which is a regulator of aldosterone secretion acting directly on adrenal glomerulosa (45). Consequently, through mineralocorticoid receptor hyperactivation, aldosterone may promote adipose tissue differentiation, growth and chronic inflammation. Overall, these mechanisms result in a detrimental “loop” which is independent from the classical aldosterone secretion stimuli (i.e sympathetic nervous system, renin-angiotensin-aldosterone system, serum potassium and sodium) may contribute to detrimental effects on the cardiovascular system (46, 47).

### Effects on Daytime Systolic and Diastolic Blood Pressure

The majority of meta-analysis focusing on hypertension outcomes following bariatric surgery have described that bariatric surgery (mostly RYGB) offered superior results in terms of hypertension control compared to non-surgical methods such as diet and nutritional follow-up by obesity specialists (17–20, 48). We observed a similar reduction in SBP and DBP in both groups without significant difference in BP reduction between hypertensive and non-hypertensive patients. This observation remained true after 3 and 12 months despite a major difference in weight loss between groups. This lack of correlation between BP reduction and weight loss is in line with other studies that emphasized the modest change in BP despite very significant weight loss after bariatric surgery (49). However, it may be plausible that a more important reduction of BP in the RYGB, especially in the hypertensive patients, was masked by the short duration of the follow-up. Indeed, most of the studies that analyzed the impact of bariatric procedure on BP control had longer follow-ups (from 2 to 5 years). This interpretation is reinforced by the fact that in the RYGB group, the decrease in BP appeared only after 12 months. Finally, this lack of correlation between weight loss and the decrease in BP may be the consequence of the small sample size that masked the true effect of bariatric-induced weight loss on BP reduction.

### Effects on Creatinine Clearance, Inulin Clearance, Albuminuria, and Electrolytes Excretion

The majority of studies observed that GFR decreased after bariatric surgery in subjects with hyperfiltration but increased in subjects with renal failure (50, 51). To our knowledge, a comparison of diet-induced and bariatric-induced weight loss on renal function has not been published yet. It is important to keep in mind that the results of the majority of these studies need to be interpreted with caution as most of them used formulas that estimate GFR based creatinine (e.g MDRD and CKD-EPI). Indeed, these equations do not provide accurate estimation of the true GFR in obese patients (34).

Initially, glomerular hyperfiltration was present in 44% of patients in the RYGB group and 83% in the control group. This difference between groups was not statistically significant and was interpreted because of the small sample size. None of our patients had criteria for renal impairment (eGFR < 60 ml/min/1.73 m^2^) at baseline. However, we observed a continuous reduction of albuminuria and renal clearance (whether based on inulin or creatinine clearance) during the follow-up. This decrease in mGFR and albuminuria in the RYGB was not different between groups. Moreover, we found that using inulin clearance a large proportion of the patients from the RYGB group reached criteria of a mild chronic kidney disease corresponding to a mild reduction of GFR [stage G2, 60-89 ml/min according to KDIGO 2012 (52)] with a mean inulin clearance of 83 ± 36 ml/min after 3 months and 68 ± 20 ml/min after 12 months of follow up. We think that these results should not necessarily be interpreted as a pernicious effect of bariatric surgery on kidney function but rather as a pre-existing mild kidney failure that was masked by the hyperfiltration and that is revealed after drastic weight loss and its accompanying reduction of glomerular hyperfiltration. Also, we observed that the decrease in renal clearance was not correlated with a higher excretion of oxalate, a finding that has been described previously (53, 54). On the contrary, we found a positive correlation between urinary oxalate excretion and renal clearance, reflecting the fact that the higher the GFR, the higher the oxalate excretion. One of the explanations of the divergence of these results with the existing literature might be the duration of the follow-up and the methods used to measure or estimate GFR. Indeed, a longer follow-up would highlight the impact of high oxalate excretion and oxalate deposition in the kidneys on the reduction of renal clearance. Overall, we found a decrease in mGFR indicating that glomerular hyperfiltration was significantly reduced. However, whether this reduction of filtration rate could reveal long term renal function stabilization, or the beginning of hyperoxaluria-induced renal impairment remains to be evaluated in larger studies with a longer follow-up.

To our knowledge, there is very limited data in the literature regarding electrolytes urinary excretion following bariatric surgery, excepted for oxalate and citrate excretion. We observed a subacute decrease in the excretion of all the electrolytes 3 months after the surgery, excepted for oxalate, magnesium and citrate. Between 3 to 12 months, daily urinary excretion tended to increase again but remained lower than the baseline daily excretion level. The first reason for this observation may be the drastic reduction of dietary intake following the first 3 months of RYGB. After the first 3 months, the tendency of the daily excretion to increase might be reflecting some kind of renal adaptation toward a new homeostasis, even though the baseline daily excretion level was not reached after one year. Of note, there was no differences in electrolytes excretion profile across the study when adjusted for weight loss. The main mechanism responsible for hyperoxaluria after bariatric surgery is the complexation of unabsorbed fatty acids to calcium in the intestinal lumen, which increases intestinal oxalate absorption. However, a decrease in calcium intake (reflected by lower calcium excretion observed at 3 and 12 months), could also have contributed to increased oxalate absorption.

Several limitations have to be mentioned in our study. First, the study was non-randomized, which could have introduced a selection bias. However, the characteristics of the two groups did not differ. In addition, the statistics used allowed us to take repeated measures in time and to adjust for some confounding factors. Finally, the sample size of our study was a relatively small.

To conclude, our findings suggest that RYGB-associated weight loss decreases PAC secretion independently of plasma renin activity or sodium excretion. Whether the decrease in PAC and leptin limits target organ damage in the long term remains to be determined.

## Data Availability Statement

The raw data supporting the conclusions of this article will be made available by the authors, without undue reservation.

## Ethics Statement

The studies involving human participants were reviewed and approved by Commission Cantonale d’éthique de la Recherche sur l’être Humain, Canton de Vaud, Switzerland, http://www.cer-vd.ch. The patients/participants provided their written informed consent to participate in this study.

## Author Contributions

MB: Data analysis, interpretation of data, statistical analysis, and drafting of manuscript. NV: Acquisition of data and study design. MM and EG: Laboratory and blood samples analysis. MF: Statistical analysis. LF: Participation to patient’s recruitment and contribution to the writing of the manuscript. OB: Critical revision of the manuscript for important intellectual content. GW: Study concept and design, conducting and study supervision, and participation to the writing of the manuscript. All authors contributed to the article and approved the submitted version.

## Funding

This study was funded by a grant from the Swiss National Foundation no 32003B_149903.

## Conflict of Interest

The authors declare that the research was conducted in the absence of any commercial or financial relationships that could be construed as a potential conflict of interest.

## Publisher’s Note

All claims expressed in this article are solely those of the authors and do not necessarily represent those of their affiliated organizations, or those of the publisher, the editors and the reviewers. Any product that may be evaluated in this article, or claim that may be made by its manufacturer, is not guaranteed or endorsed by the publisher.
